# Fatigue insights from walking tests in spinal cord injury and multiple sclerosis individuals

**DOI:** 10.1038/s41598-024-55238-8

**Published:** 2024-02-27

**Authors:** Sara Fernández-Canosa, Angela Brocalero-Camacho, Alicia Martínez-Medina, Eva Díez-Rodríguez, Pablo Arias, Antonio Oliviero, Vanesa Soto-León

**Affiliations:** 1 FENNSI Group, Hospital Nacional de Parapléjicos, SESCAM, 45004 Toledo, Spain; 2 Asociación de Esclerosis Múltiple de Toledo (ADEMTO), 45007 Toledo, Spain; 3 Department of Physiotherapy, Medicine and Biomedical Sciences and INEF Galicia, NEUROcom (Neuroscience and Motor Control Group) and Biomedical Institute of A Coruña (INIBIC), Universidade da Coruña, 15179 A Coruña, Spain; 4 Advanced Rehabilitation Unit, Hospital Los Madroños, 28690 Brunete, Spain

**Keywords:** Neurological disorders, Multiple sclerosis, Spinal cord diseases, Neuroscience, Motor control, Fatigue

## Abstract

In the last decade, fatigue in clinical populations has been re-conceptualized, including dimensions such as perceived fatigue (trait and state fatigue) and fatigability. The aim of this study was to evaluate different expressions of fatigue in Spinal Cord Injury (SCI) and Multiple Sclerosis (MS) participants compared to able-bodied controls, during activities of daily living, especially during gait. A total of 67 participants were included in this study (23 with SCI, 23 with MS, and 21 able-bodied controls). All participants performed two functional tests (6-Minute Walk Test and 10-Meter Walk Test) and they completed the Fatigue Severity Scale (FSS). The rate of trait fatigue was different between groups, with MS participants showing the highest rate. Moreover, scores on functional tests and state fatigue were different between groups after the tests. Our results indicate that trait fatigue and state fatigue in individuals with SCI and MS are different with respect to able-bodied population. Both SCI and MS groups experienced more trait fatigue than control group in daily life. In addition, walking tasks produced similar levels of state fatigue between healthy people and patients with MS/SCI. However, these tests induced longer-lasting levels of state fatigue in the patients.

## Introduction

Fatigue is a universal experience and refers to the difficulty in initiating or sustaining voluntary activities, although a universally accepted definition of fatigue has not been reached. However, the knowledge about pathophysiology of fatigue is limited^1,2^. In healthy subjects, fatigue is a physiological reaction to a prolonged and/or intense physical activity. Fatigue is task-dependent, it reduces with rest and usually does not interfere with daily activities^3^. However, individuals with certain pathologies describe fatigue as an overwhelming sense of tiredness at rest, exhaustion with activity, lack of energy that precludes daily tasks, inertia, or loss of vigor^4^.

The prevalence of fatigue is high in many neurological illnesses. It occurs in over 50% of community-dwelling people with Spinal Cord Injury (SCI)^5^. Fatigue is also the most common symptom in Multiple Sclerosis (MS), affecting up to 90% of patients during their life. Two-thirds of patients describe it as their most disturbing symptom^6^. In these patients, the effects of fatigue on function may create an additional barrier to community reintegration; therefore, fatigue needs to be better understood to improve treatment in these patients^7^.

Scientific research on fatigue includes several terminologies used inconsistently in the literature^8^. Fatigue is a widely used term that refers to several meanings, causalities, and dimensions: amongst the latter “the perception of fatigue” and “performance fatigability”^9^. The perception of fatigue is divided into "state fatigue" and "trait fatigue"^10^. “State fatigue” has been defined as a person’s self-reported transient sensation of weariness or "subjective feeling" of diminished capacity during or right after exercise^9,11,12^. It has been measured by an analog of the Borg scale to assess state fatigue right after exercise^9,13^. "Trait fatigue" refers to a frequent, prolonged sensation of fatigue experienced during the preceding several days and can be assessed by the Fatigue Severity Scale (FSS)^14,15^. This questionnaire measures the impact of fatigue on functional disability and has been shown to be internally consistent, and sensitive to clinical changes^7,16^.

On the other hand, fatigability is an objective decline in performance (force development, power, speed, reactivity, or accuracy) observed during cognitive or motor tasks^2,9,17^. What remains unclear is whether fatigue (trait fatigue and state fatigue) and performance fatigability are similarly affected in MS and SCI^18,19^. The aim of this study was to evaluate perceived fatigue and performance fatigability during a functional daily task (walking) in two pathological (SCI & MS) and able-body groups of participants. We hypothesize that patients would have greater trait fatigue, greater fatigability while walking as well as larger levels of state fatigue than able controls.

## Methods

### Design

Our study was a prospective study that evaluated fatigue in individuals with SCI, MS, and able-bodied individuals.

### Study population

A total of 67 individuals participated in this study. Individuals with SCI and able-bodied controls were recruited from the Hospital Nacional de Parapléjicos of Toledo (HNP) and individuals with MS from Asociación de Esclerosis Múltiple of Toledo (ADEMTO) from March 2021 to June 2022. Our inclusion criteria for the participants with SCI and MS included age over 18 years, ability to walk at least 10 m, and preserved capacity to understand the instructions of the study. Exclusion criteria were major psychiatric disorder, presence of other neurological illness, pregnancy, lactation period, and—for MS patients—recent relapse (< 3 months). All procedures conformed to the principles outlined in the Declaration of Helsinki and informed consent was obtained from each participant agreeing to be enrolled in this study. The procedure was approved by our local ethics committee of Hospital Universitario de Toledo.

### Clinical assessments

Demographic and clinical characteristics were collected from all participants by the same expert physician (A.B.C). The disease severity of SCI and MS was evaluated using the International Standards for Neurological Classification of SCI (ISNCSCI) ASIA and the Impairment Scale and the Expanded Disability Status Scale (EDSS), respectively^20,21^. These assessments were done by the same expert physiotherapist (S.F.C).

The ISNCSCI is a standardized examination that classifies injuries as a complete or incomplete SCI. A complete SCI is defined as the absence of motor and sensory functions below the lesion level (AIS: Grade A) and incomplete injuries are defined as those with some degree of retained motor or sensory function below the site of injury (AIS: B to E)^20^.

For MS patients we used the EDSS, which is a clinician-administered assessment scale to describe disease. The rating system ranges from 0 (normal neurological status) to 10 (death due to MS) in 0.5 increments interval^21^.

Trait fatigue was assessed in all the participants using the FSS, which was determined before any functional test. The FSS is a 9-item questionnaire that assesses the severity of fatigue symptoms. It requires participants to choose the degree of agreement on a 7-point Likert scale, ranging from strongly disagree (number 1) to strongly agree (number 7). The FSS assessment is based on patients’ perceptions during the preceding week. The overall score is determined as the average score from the 9 items^7,14^. The criterion used to determine clinically significant fatigue (CSF) was a score greater than or equal to 4 in the FSS (considering the mean scores from the nine items)^22^.

### Functional tests

After the clinical evaluation and the demographic interview, we performed the functional motor tasks to evaluate walking ability and motor fatigability. For this purpose, we used the 6-Minute Walk Test (6MWT)^23^ and the 10-Meter Walk Test (10MWT)^24,25^.

The 6MWT evaluates walking ability and can be used to measure changes in the walking pattern that may appear during a normal day for MS and SCI^9,17^. This test has previously been used to evaluate fatigability, determined by a decrease in speed during the 6MWT in different neurological diseases^23,26–29^. After the test, subjects rate their current state fatigue and their dyspnea on a numerical rating scale (NRS) from 0 to 10^30,31^. We also collected data on their cardiorespiratory function before and after the test to assess their basal state and reactivity to exertion.

Moreover, the 10MWT evaluates the walking speed in patients with neurological disorders^24,25^ The 10MWT is used for obtaining information about walking speed. This is a well-recognized parameter associated with survival, frailty, and functional capacity^32–34^. Then, we assessed the state fatigue again, following this test.

6MWT and 10MWT were carried out by all participants, and these were recorded by video camera. All participants performed the tests following the same procedure. Both tests were done on a flat and smooth floor, with two cones placed on each end. Participants rested in a seating position for 15 min before starting to walk. 6MWT was performed first. This test measures the maximum distance (in meters) walked for 6 min^35,36^. The test was conducted on a 20-m corridor. Participants were instructed to walk as fast as possible at a safe speed. They were encouraged with standard phrases every minute^37^. They were permitted to rest if they needed but the stopwatch was not paused anytime. The total distance covered for each participant was calculated and recorded. Before and immediately after (in the following 2 min) the 6MWT, we acquired the following parameters: heart rate (HR), blood pressure (BP), and oxygen saturation (OS). Immediately after these cardiovascular and respiratory measures, the dyspnea rating and the state fatigue were obtained. HR and BP were measured with a digital tensiometer, a pulse oximeter was used to measure OS, and a NRS was used to measure dyspnea and state fatigue. Participants were asked to rate their current level of fatigue state using a single question “How physically tired are you, right now?”. They were also asked to rate their current level of dyspnea using a single question “How severe would you rate your difficulty in breathing, right now?”. Both questions were rated on a NRS ranging from 0 to 10.

10MWT measures the time (in seconds) needed to walk 10 m at maximum speed. We did the test with dynamic flying, with 2-m acceleration at the start and 2-m of deceleration at the end^25,38^. After this walking test, state fatigue was again measured.

All participants performed the 10MWT after the 6MWT with 15 min rest between tests. The resting period of 15 min is considered adequate recovery time in walking tests^37^.

**Figure 1:**
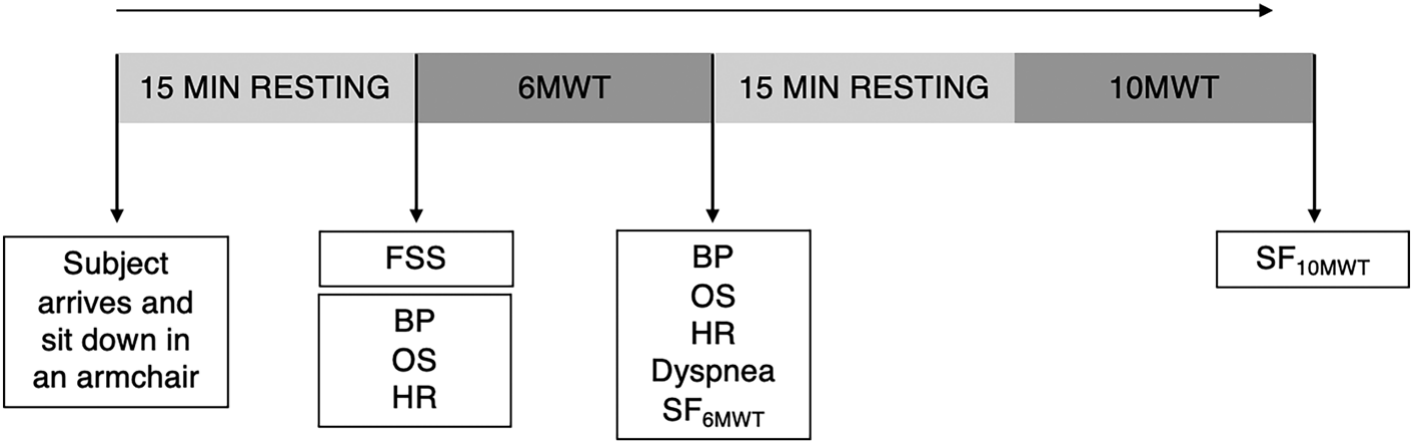
Schematic description of the whole procedure. The protocol used to all the population included. FSS: Fatigue Severity Scale; BP: Blood Pressure; OS: Oxygen Saturation; HR: Heart Rate; SF_6MWT_: State Fatigue after 6-Minute Walk Test; SF_10MWT_: State Fatigue after 10-Meter Walk Test.

Schematic description of the whole procedure is provided in Fig. 1.

### Data analysis

We present continuous variables as the mean and standard deviation and discrete variables as the median and interquartile range. Categorical variables are expressed as counts and percentages.

Parametric test (One-way ANOVA) and nonparametric univariate (Chi-squared) testing were used to compare demographic variables between three groups.

FSS was compared between groups with one-way ANOVA. If there was a significant difference, we used the standard Bonferroni post-hoc correction.

Scores on 10MWT, 6MWT, State Fatigue after 6MWT (SF_6MWT_), State Fatigue after 10MWT (SF_10MWT_), and dyspnea were compared between participants with SCI, MS, and able-bodied controls with a Kruskal–Wallis test. In case of significant effects, we used Dunn Bonferroni post-hoc testing for pair-wise comparison.

Respiratory and cardiovascular data were analyzed separately, systolic BP, diastolic BP, and HR were compared between groups using one-way ANOVA. In case of a significant main effect, we used the Bonferroni post-hoc analyses. OS was compared between groups with Kruskal–Wallis test, Dunn post-hoc was used in case of significative differences. In addition, we compared the clinical data before and after the 6MWT with the paired samples t-test in all the groups separately. Furthermore, to compare the effects the effects of the 6MWT on these parameters the percentage of change (post/pre*100) was calculated individually and then compared between groups.

Finally, to analyze 6MWT fatigability, we calculated the distance walked at participants’ fastest safe speed in the first minute (6MWT_D1_) and in the last minute (6MWT_D6_) for all individuals (controls, SCI and MS group). These analyses were done fitting the data (time required to walked 40 m or 1 lap) in two straights (one of them for the first minute and the other one for the last minute) and the adjusted data were used to calculate the distance in both minutes. Therefore, the distance only can be calculated if participant did at least 1 lap (n = 62). In each group, the paired samples t-test was used to test for the difference between the 6MWT_D1_ and 6MWT_D6_.

The normality of the data was verified prior to analysis with the Shapiro–Wilk test. All statistical analyses were performed with the software JASP (Version 0.16.1). Differences were considered significant at *p* < 0.05.

## Results

Data from 67 individuals were analyzed: 23 with SCI, 23 with MS and 21 controls. Demographic and clinical data from the participants are reported in Supplementary Table 1.

There were no statistical differences between SCI, MS, and controls regarding gender proportion (χ^2^ = 4.295, *p* = 0.117) and age (F_2,64_ = 2.876, *p* = 0.064). All patients with SCI had incomplete injuries (13% AIS C and 87% AIS D). Among them, 61% had cervical lesions, 30.4% had thoracic lesions, and 8.6% had lumbar lesion. Regarding patients with MS, the median EDSS obtained was 4.5 and the Interquartile Range was 3.5 and the most predominant type of MS was relapsing–remitting (RRMS) with 60.9%.

**Figure 2:**
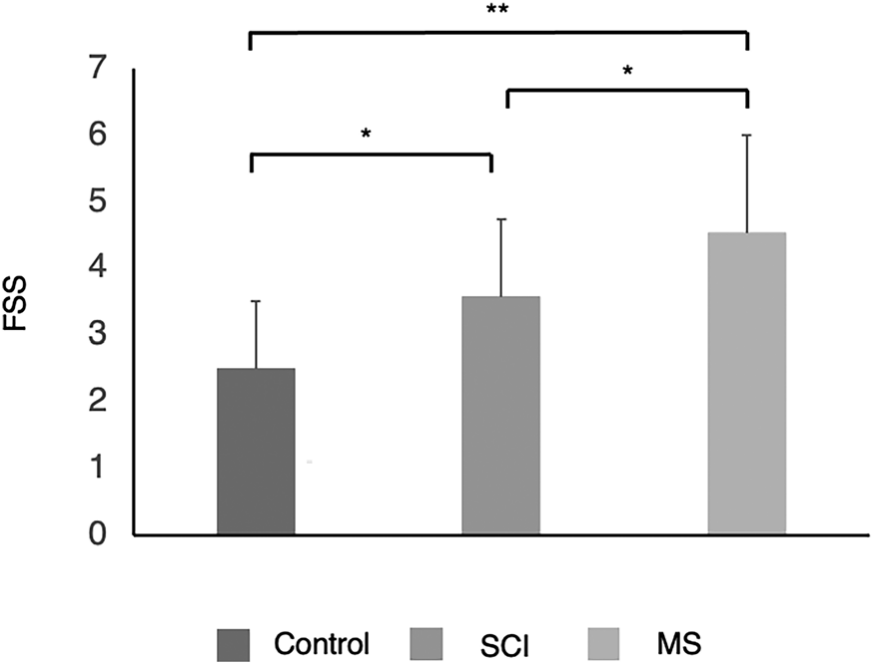
FSS group comparison. Group comparison of the scores of the FSS. SCI: spinal cord injury; MS: multiple sclerosis; FSS: scores of Fatigue Severity Scale. **p < 0.001; *p < 0.05.

The prevalence of CSF was 34.8% in SCI group, 73.9% in MS group and 9.5% in able-bodied controls. There were significant differences in scores of FSS between groups, shown in Fig. 2, ANOVA (F_2,64_ = 14.822, *p* < 0.001). Bonferroni post-hoc tests that MS had higher scores of FSS than SCI and control group (*p* = 0.03; *p* < 0.001, respectively), also, SCI group had higher FSS than controls (*p* = 0.017).

### Group comparisons: functional tests

#### 6MWT and 10MWT

6MWT and 10MWT results are summarized in Table 1.

**Table 1:** Results of *6MWT and 10MWT*. Results for univariate test: Kruskal–Wallis test. Differences between results of 6MWT and 10MWT between groups (control, SCI and MS).

Parameters	CONTROL	SCI	MS	*p* value	Effect size (η^2^ [H])
*N*	21	23	23		
6MWT_meters_ (M [IQR])	693.1 [121.9]	160 [167.7]	302.6 [202.3]	< 0.001**	0.638
SF_6MWT_ (M [IQR])	2 [3]	3 [4]	3 [3.5]	0.061	0.056
Dyspnea (M [IQR])	0 [1]	3 [3.25]	3 [3.5]	< 0.001**	0.221
10MWT_seconds_ (M [IQR])	4.18 [0.48]	13.06 [13.05]	9.55 [5.41]	< 0.001**	0.647
SF_10MWT_ (M [IQR])	0.5 [0.5]	3 [2.5]	3 [5]	0.013*	0.118

N: Number of participants; SCI: Spinal Cord Injury; MS: Multiple Sclerosis; 6MWT_meters_: 6-Minute Walk Test Distance (meters); X̄: Mean; SD: Standard Deviation; SF_6MWT_: State Fatigue after 6-Minute Walk Test; M: Median; IQR: Interquartile Range; 10MWT_seconds_: 10-Meter Walk Test Time (seconds); SF_10MWT_: State Fatigue after 10-Meter Walk Test **p < 0.001; *p < 0.05.

**Figure 3:**
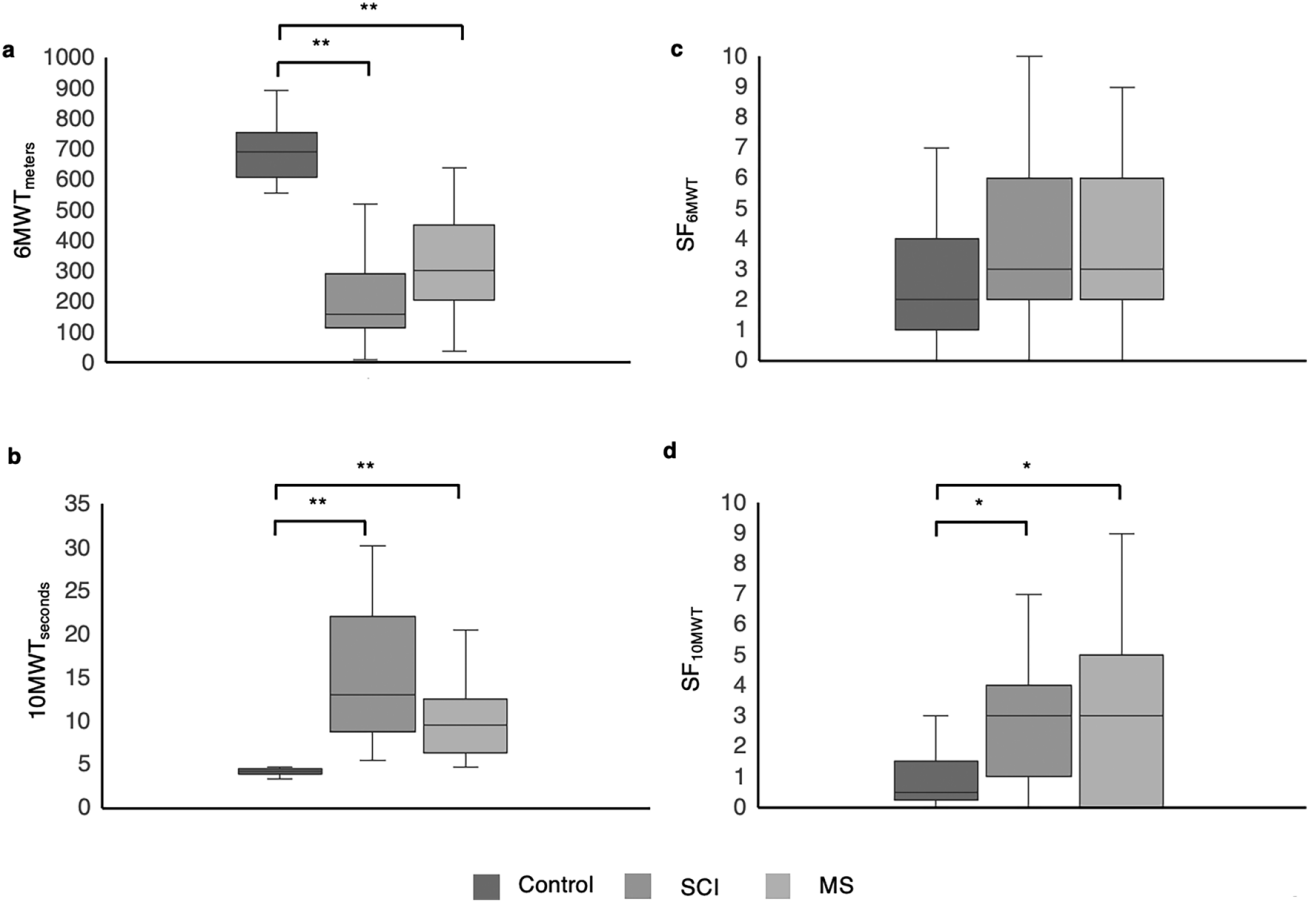
Comparison of scores of variables related to fatigue. (**A**) Group comparison of the distance (meters) of 6MWT; (**B**) group comparison of the time (seconds) of 10MWT; (**C**) group comparison of Rate Perceived Exertion Test after 6MWT; (**D**) group comparison of Rate Perceived Exertion Test after 10MWT. SCI: Spinal Cord Injury; MS: Multiple Sclerosis; 6MWT_METERS_: 6-Minute Walk Test Distance (meters); 10MWT_SECONDS_:10-Meter Walk Test Time (seconds); SF_6MWT_: State Fatigue after 6-Minute Walk Test; SF_10MWT_: State Fatigue after 10-Meter Walk Test. **p < 0.001; *p < 0.05.

Results of 6MWT and 10MWT were different between groups (H = 42.88, *p* < 0.001; H = 42.08, *p* < 0.001, respectively). Distance walked by the control group in 6MWT was significantly larger than distance walked by SCI and MS groups (*p* < 0.001). However, distance of the MS group was not longer than distance of the SCI group (*p* = 0.056) (Fig. 3a).

Time used in 10MWT was lower in controls than in other groups (*p* < 0.001), but no difference was found between SCI and MS groups in time (*p* = 0.173) (Fig. 3b). Visual inspection of the data suggests this lack of significance is due to the greater variability of the data in SCI group compared to the MS. Levene’s Test confirmed this variability (*p* = 0.035).

Kruskal–Wallis analysis did not reveal significant differences between groups for SF_6MWT_ (H = 5.588, *p* = 0.061) (Fig. 3c). Dyspnea after 6MWT was significantly different among groups (H = 16.155, *p* < 0.001), being higher in both groups of individuals with SCI and MS than controls (*p* = 0.003 for MS and *p* < 0.001 for SCI). However, SCI and MS did not show differences in dyspnea (*p* = 0.192).

Similarly, SF_10MWT_ was different between groups (H = 9.534, *p* = 0.009) (Fig. 3d). Dunn post-hoc testing found that SCI group and MS group had higher scores of SF_10MWT_ compared to the control group (*p* = 0.004; *p* = 0.036, respectively). MS and SCI did not show differences in SF_10MWT_ (*p* = 0.17).

**Figure 4:**
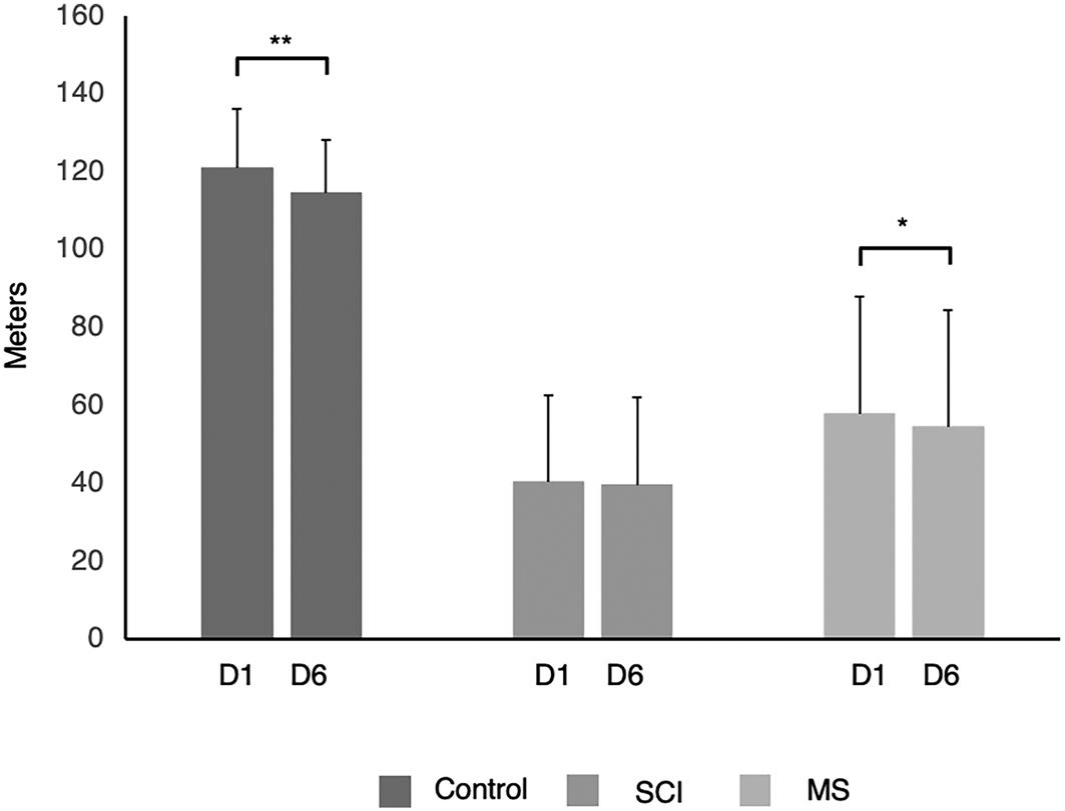
Fatigability during the 6MWT: comparison of the first minute with the sixth minute of the test. Comparison between the 6MWT_D1_ with 6MWT_D6_ in each group: control, SCI and MS groups. SCI: Spinal Cord Injury; MS: Multiple Sclerosis; 6MWT_D1_: Distance first minute of the 6MWT; 6MWT_D6_: Distance sixth minute of the 6MWT. **p < 0.001; *p < 0.05.

We conducted a further analysis of the distance covered during the 6MWT. 6MWT_D1_ and 6MWT_D6_ were compared in all groups. Paired samples t-test revealed statically significant differences between distances in control group (t = 6.792, *p* < 0.001) and in MS group (t = 2.111, *p* = 0.048), indicating that both groups of participants decreased their 6MWT_D6_ compared to 6MWT_D1_. However, SCI group did not show differences between 6MWT_D1_ and 6MWT_D6_ (t = 0.846, *p* = 0.408) (Fig. 4).

### Cardiovascular and respiratory tests

These data were obtained before (at rest) and immediately after the effort to complete the 6MWT. At rest, systolic BP (F_2,64_ = 0.519, *p* = 0.598, η2 = 0.016), diastolic BP (F_2,64_ = 1.31, *p* = 0.277, η2 = 0.039) and HR (F_2,64_ = 2.817, *p* = 0.067, η2 = 0.081) were similar in all groups. On the other hand, OS showed different values between groups (H = 22.26, *p* < 0.001, η2[H] = 0.375), with the control group having higher values of OS than both patient groups (*p* < 0.001). SCI and MS groups obtained similar values of OS before the test (*p* = 0.368).

Moreover, we analyzed the cardiovascular and respiratory parameters before and after 6MWT. The control group showed higher systolic BP (t = −6.114, *p* < 0.001, d = −1.334) and HR (t = −7.362, *p* < 0.001, d = −1.606) and lower values of OS (t = 2.553, *p* = 0.023, d = 0.659) after 6MWT. Systolic BP (SCI: t = −3.77, *p* = 0.001, d = −0.786; MS: t = −2.990, *p* = 0.007, d = −0.623), and HR (SCI: t = −3.673, *p* = 0.001, d = −0.766; MS: t = −2.312, *p* = 0.031, d = −0.482) were significantly higher both in SCI and MS after 6MWT (Table 2). Only in SCI group, diastolic BP (t = −2.144, *p* = 0.043, d = −0.447) was higher after the test (Control group and MS: all *p* > 0.5). OS was similar before and after 6WMT in both individuals with SCI and MS (*p* > 0.08).

**Table 2:** Cardiovascular and respiratory tests. Results for univariate test: One-Way Anova or Kruskal–Wallis test, Paired Samples T-test. Cardiovascular and respiratory data pre 6MWT and post 6MWT. In data of post 6MWT are in bold the differences that are statically significant (paired t test) comparing pre and post 6MWT. Percentual change of the cardiovascular and respiratory data after 6MWT.

Parameters	CONTROL	SCI	MS	*p* value	Effect size
*N*	21	23	23	Group analysis	η^2^[H]/η^2^
At rest					
BP_systolic_ (X̄ ± SD)	115.5 ± 16.2	111.7 ± 17	110.4 ± 18.2	0.598	0.016
BP_diastolic_ (X̄ ± SD)	78.9 ± 14	73 ± 10.9	76.7 ± 11.9	0.277	0.039
OS (median [IQR])	99 [1]	97 [1]	96.5 [1]	< 0.001	0.375
HR (X̄ ± SD)	71 ± 7.9	78.7 ± 13	73.9 ± 10.8	0.055	0.081
Post 6MWT					
BP_systolic_ (X̄ ± SD)	140 ± 21.1**	123.7 ± 19.5*	117.5 ± 18.4*		
BP_diastolic_ (X̄ ± SD)	80.4 ± 9	76.5 ± 12*	77 ± 11.7		
OS (X̄ ± SD)	98 [1] *	97 [2]	97 [1]		
HR (X̄ ± SD)	98.8 ± 17.2**	85 ± 11*	78 ± 14*		
Percentual change after 6MWT (post/pre*100)					
BP_systolic_ (% ± SD)	122.2 ± 17.2	111.6 ± 15.6	107.2 ± 10.5	0.004	0.159
BP_diastolic_ (% ± SD)	103.73 ± 13.57	105.4 ± 11.56	100.8 ± 7.85	0.213	0.030
OS (% ± SD)	99.4 ± 0.9	100.5 ± 1.4	100.3 ± 1.3	0.026	0.126
HR (% ± SD)	140.4 ± 26.2	109.4 ± 12.8	105.5 ± 7.9	< 0.001	0.441

N: Number of participants; X̄: Mean; SD: Standard Deviation; SCI: Spinal Cord Injury; MS: Multiple Sclerosis; BP_systolic_: Systolic Blood Pressure; BP_diastolic_: Diastolic Blood Pressure; OS: Oxygen Saturation; IQR: Interquartile Range; HR: Heart Rate; %: percentual change after 6MWT (post/pre*100). **p < 0.001; *p < 0.05.

The percentage changes after 6MWT were compared among the three groups (Table 2) This analysis confirmed that the control group had a significantly higher increment of systolic BP and HR and more reduction of OS (all *p* < 0.05).

## Discussion

Fatigue is a common symptom in MS and SCI and its assessment and treatment are poorly understood by physicians and therapists. The aim of this study was to evaluate the perceived fatigue and motor fatigability in individuals with MS and SCI and to compare them to able-bodied participants.

Our findings indicated that perceived fatigue in daily life is higher in MS group than SCI and control groups. Additionally, the SCI group experiences more fatigue in their daily life compared to the control group. This result coincides with our hypothesis that patients would have greater fatigue in their daily life than controls. The prevalence of fatigue in neurological disorders has been widely studied^39–43^. There is evidence for 50% to 70% prevalence of fatigue in patients with MS^39–41^ and from 19 to 57% in SCI population^42,43^. This variability across different studies may be caused by the different demographic and clinical characteristics of the patients. Our result was similar to data previously reported^40,44^. In our study, we confirmed that trait fatigue (perceived fatigue) is higher in individuals with SCI and MS compared to able-bodied controls (and higher in MS than in SCI). Furthermore, we found that the prevalence of CSF was higher in individuals with MS (~ 74%) and individuals with SCI (~ 35%).

Furthermore, our results of the functional tests were different among groups. As expected, control group obtained the best scores both in 6MWT and 10MWT compared to both individuals with SCI and MS. Regarding SF_6MWT_, three groups obtained similar values. As far as SF_10MWT_, the control group was not fatigued at all, while both SCI and MS groups showed moderate state fatigue again.

We are unsure as to why patient groups have higher SF_10MWT_. One possibility is that the 10MWT produces more state fatigue in patient groups than in able-bodied controls because any effort may higher levels of perceived fatigue in patients. An alternative explanation could be that the controls have preserved recovery capacity, which appears to be impacted in the patient groups, therefore SF_10MWT_ is affected by the fatigue caused by the 6MWT.

Both individuals with SCI and MS had more dyspnea than Controls after the 6MWT. This may be because patients may have cardiopulmonary pathology and it is an important factor for patient functional capacity in daily living (see below). High scores of dyspnea have already been described in SCI population during their daily activities^45^. These values of dyspnea in patient populations can increase their fatigability and their perceived fatigue, indicating that their fatigue is more multidimensional than fatigue in controls. There are studies that confirm this relationship. Devasahayam et al. showed in their study that the oxygen cost is significantly higher in patients with MS compared to controls in daily activities and that this oxygen cost strongly correlates with task-induced perceived fatigue^46^. Also, Jensen et al. found significant associations between dyspnea and fatigue in chronic SCI^47^.

Regarding respiratory and cardiovascular tests, there were differences between groups in the OS at rest. These differences may arise from different factors, including features of, neurological disease, pharmacological treatments, reduced occupational and physical activities and poor sleep quality. At least in part, these alterations may explain worse performance of patients and higher state fatigue after recovery^17^. We also found differences between groups before and after the 6MWT. As expected, control group increased their values of HR and BP and decreased the OS after the test. Halliday et al. obtained similar results; they found an increase of HR after 6MWT in healthy population^48^. They also found a positive strong correlation between this increase and the number of meters covered during the 6MWT^48^. The cardiovascular change was much less evident in both individuals with SCI and MS. This can be due to their pathologies causing also autonomic dysfunction, or other reasons such as, for example, pharmacological therapy, so that patients’ performance is worse and the state fatigue and dyspnea higher^49^. Alternatively, it might be that patient’s maximal effort is not enough to produce changes in cardiovascular and respiratory responses after walking. To test fatigability while walking during the 6MWT, like others^23,26–29^, we compared the 6MWT_D1_ and 6MWT_D6_ performances. We found that control and MS groups decreased their speed from the first minute to the sixth minute. On the contrary, the SCI group maintained their speed during 6MWT. We do not have a clear explanation for this difference between SCI and the other groups. We can suggest that individuals with SCI are slower than individuals with MS and control individuals and that, for this reason, their velocity is not decaying. Furthermore, we cannot exclude changes in gait quality (not assessed in our experiments)^50^. That is, individuals with SCI may change their kinematics during the walking test and in this way, they may not decrease their speed. Moreover, it is possible that spasticity decreases over distance (and may compensate fatigue). Hitherto, this explication is unlikely, as individuals with MS also suffer from spasticity.

Between-group differences in the cardiovascular response suggest that there are substantial differences in how both able-bodied and individuals with SCI and MS deal with the 6MWT. Whether this is the cause or the effects, it seems clear that individuals with SCI deal differently with the 6MWT and both individuals with SCI and MS have more dyspnea and have higher SF after 10MWT than controls.

In summary, our results seem to indicate that trait fatigue and state fatigue of individuals with SCI and MS are different with respect to able-bodied population. Both SCI and MS patients perceive larger levels of trait fatigue than control group in their daily life. In addition, walking tasks produced similar levels of state fatigue between healthy people and patients with MS/SCI. However, these tests induced longer-lasting levels of state fatigue in the patients. Cardiovascular and respiratory factors need further study to determine whether they are the cause or the effect of higher fatigability at rest or after a physical effort.

### Limitations

A NRS ranging from 0 to 10 was used to assess state fatigue. Immediately after performing the task, the participants were asked to answer a single question regarding their fatigue level. This assessment scale is similar to the Borg scale but has not been validated for this purpose.

### Supplementary Information

Supplementary Information.

## Data Availability

Data are available upon reasonable request from Hospital Nacional de Parapléjicos de Toledo by contacting the author V.S-L.
