# Supplementary material for: Fatigue insights from walking tests in spinal cord injury and multiple sclerosis individuals
Source: Sci Rep. 2024 Feb 27;14:4761. doi: 10.1038/s41598-024-55238-8 (PMC10899186; doi:10.1038/s41598-024-55238-8)
Supplement: Supplementary file 1 — Supplementary Information. [file 41598_2024_55238_MOESM1_ESM.pdf]

SUPPLEMENTARY INFORMATION

Fatigue in spinal cord injury and multiple sclerosis individuals: insights from walking tests

Sara Fernández-Canosa1, Angela Brocalero-Camacho1, Alicia Martínez-Medina2, Eva Díez-Rodríguez1, Pablo Arias3, Antonio Oliviero1,4*, Vanesa Soto-León1*

1 FENNSI Group, Hospital Nacional de Parapléjicos, SESCAM, 45004 Toledo, Spain.

2 Asociación de Esclerosis Múltiple de Toledo (ADEMTO), 45007 Toledo, Spain.

3 Universidade da Coruña, Neuroscience and Motor Control Group (NEUROcom), Department of Sport and Physical Education, Department of Physiotherapy, Medicine and Biomedical Sciences, INEF-Galicia, Institute of Biomedical Research of A Coruña, 15179 A Coruña, Spain.

4 Advanced Rehabilitation Unit, Hospital Los Madroños, 28690 Brunete, Spain.

Supplementary tables

Supplementary Table 1. ***Demographic and clinical data***

**Parameters**	**CONTROL**	**SCI**	**MS**	***p* value**
N	21	23	23	
Demographic				
Age ($\bar{X}$ ± SD years)	41 ± 14.39	49.4 ± 14.68	49.3 ± 10.2	0.064
Sex (Female/Male)	10/11	8/15	16/7	0.117
Clinical data				
AIS (n [%])				
C	N/A	3 [13]	N/A	
D	N/A	20 [87]	N/A	
Level of SCI (n [%])				
Cervical	N/A	14 [61]	N/A	
Thoracic	N/A	7 [30.4]	N/A	
Lumbar	N/A	2 [8.6]	N/A	
EDSS (median [IQR])	N/A	N/A	4.5 [3.5]	
Type of MS (n [%])				
RRMS	N/A	N/A	14 [60.9]	
PPMS	N/A	N/A	3 [13]	
SPMS	N/A	N/A	6 [26.1]	
FSS ($\bar{X}$ ± SD score)	2.4 ± 1	3.57 ± 1.2	4.5 ± 1.5	<0.001
CSF (n [%])	2 [9.5]	8 [34.8]	17 [73.9]	<0.001

Results for univariate test: Chi-square test and One-Way Anova test. N: number of participants; SCI: spinal cord injury; MS: multiple sclerosis; $\bar{X}$: mean; SD: standard deviation; AIS: International Standards for Neurological Classification of SCI; EDSS: Expanded Disability Status Scale; IQR: Interquartile Range; RRMS: Relapsing-Remitting Multiple Sclerosis; PPMS: Primary-Progressive Multiple Sclerosis; SPMS: Secondary-Progressive Multiple Sclerosis; FSS: scores of Fatigue Severity Scale; CFS: clinically significant fatigue (FSS > 4).
